# Challenges and patenting strategies for Chinese herbal medicine

**DOI:** 10.1186/1749-8546-5-24

**Published:** 2010-07-16

**Authors:** Xinsheng Wang, Albert Wai-Kit Chan

**Affiliations:** 1 DeHeng Chen, LLC, 225 Broadway, Suite 1910, New York, NY 10007, USA; 2 Law Offices of Albert Wai-Kit Chan, PLLC, Whitestone, NY 11357, USA

## Abstract

Patents for Chinese herbal medicines can be difficult to obtain. When the active ingredients of an herbal formula are known, *danfang *(single herb prescriptions) is better protected with quantified composition claims. When the active ingredients are unknown, 'product by processing', 'method of processing', 'method of administration' and 'new use claims' are often powerful tools to distinguish a traditional *danfang *from 'the prior art'. Additional patents may also be filed continuously in the product development process. Existing patents for *fufang *(composite prescriptions) are primarily drafted to protect traditional herbal formulations. More efforts are needed to protect various herbal combinations and their multiple applications.

## Introduction

Keeping Chinese medicine formulae as trade secrets has led to some losses of knowledge and experience accumulated over generations. Drugs as well as dietary supplements are subject to regulations and disclosure requirements; it has become increasingly difficult for CM practitioners to keep their prescriptions and treatment methods as trade secrets. In 1993, the Chinese government issued the *Regulations on the Protection of Traditional Chinese Medicines *which, to this date, provides administrative protection to certain prescriptions and manufacturing processes [1].

For developing new drugs and improved formulations, many Chinese herbs are being studied by major pharmaceutical companies with modern technologies. These companies rely on patent to protect their intellectual properties. Due to vast philosophical differences between CM and Western medicine, there are challenges in patent applications for CM-related products. The present article discusses some of the challenges and proposes solutions to these challenges.

## Discussion

### Types of Chinese medicine prescriptions

According to Chinese medicine theory, local pathologic changes are always considered to be connected with the entire body [2]. A strategic combination of different ingredients of herbs is necessary to generate specific and synergistic effects.

In Chinese medicine prescriptions, single herb prescriptions are called *danfang *[3] whereas multiple herb prescriptions are called *fufang *[3] which are thought to synergize the therapeutic effects of different ingredients while minimize their toxicities [4]. *Fufang *is much more commonly used than *danfang *[4].

### Patenting strategies for danfang prescriptions

#### Patenting a danfang with known active ingredients

Active ingredients in a *danfang *may be chemically synthesized and their chemical structures may be further modified for improved safety and efficacy. Quinine, the first effective antimalarial drug, was originally discovered from the bark of the cinchona tree.

The traditional herbal form of the medicine may still exist even after the active ingredients have been developed into a pure-compound drug. For example, berberine is an isoquinoline alkaloid isolated from plants such as *Rhizoma Coptidis *(*Huanglian*) and *Cortex Phellodendri *(*Huangbai*). While berberine hydrochloride, documented for its strong antibiotic and anti-inflammatory effects, has been marketed as a pure-compound drug, herbal formulae comprising *Rhizoma Coptidis *are still widely prescribed, e.g. Coptis extract tablets (*Huangliansu Pian*) and Coptis teapills (*Huangliansu Wan*) with indications such as diarrhea, dysentery, indigestion and infections. As such, patents for the traditional herbal form of these products and patents for the pure chemical drugs may co-exist. Information of known active ingredients of an herbal medicine should be incorporated into its patent. Information such as whether the chemical structures are racemates or chiral enantiomers, or how the precise quantity range of the active ingredients in the herbal product can be determined by analytical techniques such as gas chromatography (GC), high performance liquid chromatography (HPLC) and mass spectroscopy (MS).

#### Patenting strategies for danfang with unknown active ingredients

While a *danfang *with unknown active ingredients can be patented, novelty plays a key role in its patent application. In terms of composition claims, *danfang *formulae are more vulnerable to 35 U.S.C. 102 or 35 U.S.C. 103 rejections than *fufang*. Most herbs with medical effects have been somewhat disclosed 'in prior art'. Such claims as 'a composition for treating X disease comprising H (a single herbal ingredient)' are almost invariably rejected for lack of novelty or inventive step although a traditional *danfang *formula may still be protected by a number of approaches including 'method of processing', 'product by processing', 'method of administration' and 'new use of the herb'. 'Product by processing' remains a powerful tool to distinguish a traditional *danfang *from 'prior art'. For example, U.S. Pat. 7,101,535 claims 'an oral composition effective against halitosis comprising an extract from Moutan bark (*Paeonia suffruticosa *Andrews) as an active ingredient'. While the claim was rejected for lack of novelty over prior patents, the 'product by processing' claim was allowed.

To obtain a patent for a commonly used herb as long as there is a novel perspective in the formula. For example, U.S. Pat. 7,575,772 is based on the 'process and composition for syrup and jam from *Luohanguo *(*Siraitia grosvenori *or *Momordica grosvenori*) which is the fruit of the plants belonging to *Curcubitaceae *family'. *Luohanguo *as a remedy for respiratory complaints (e.g. dry coughs) has been used in many Chinese medicine formulae. While the *Encyclopedia of Traditional Chinese Medicine *[5] recommends the use of *Luohanguo *as a laxative, the applicants of this patent claimed that the whole *Luohanguo *fruit may be processed as a natural sweetener. As 'prior art' recommended to remove the seeds and husks of *Luohanguo*, the applicants protected a unique aspect of the formula by claiming to process the whole fruit extract.

#### Patent protection of the development process

Companies developing a *danfang *herbal product may file patents on an on-going basis to protect various stages of the development. When the mechanism of an herbal formula has been elucidated, or when new applications have been discovered from a *danfang*, a series of patents must be filed along with the development process.

Found in northern China, *Xanthoceras sorbifolia Bunge *(*Wenguanguo*) is a species of the *sapindaceae *family. Its seeds have been used as a folk medicine to treat enuresis [6]. U.S. Pat. No. 6,616,943 was drawn to an herbal formula comprising *Wenguanguo *to prevent cerebral aging, improve memory and cerebral functions and cure enuresis and to treat frequent micturition, urinary incontinence, dementia, weak intelligence and Alzheimer's disease, autism, brain trauma, Parkinson's disease and other diseases related to cerebral dysfunction. U.S. Pat. 7,189,420 claims an extract of *Wenguanguo *husk for use as a medicine or food supplement. U.S. Pat. 7,514,412 claims novel compounds isolated from *Wenguanguo *that have anticancer activities. Those novel compounds may be either extracted from natural sources or synthesized chemically. Currently, U.S. Pat. 7,262,285 covers the derivatives of the isolated active ingredients.

### Patenting strategies for fufang prescriptions

#### Patent protection of herbal formulations

A strategy for protecting herbal formulations is to divide the compositions into several groups and (1) specify the composition with the most essential herbs in the independent claim; (2) specify the compositions with additional herbs as dependent claims. For example:

Claim 1: A composition for treating disease X comprising H1, H2 and H3.

Claim 2: A composition of claim 1, wherein the composition further comprises H4.

Claim 3: A composition of claim 1, wherein the composition further comprises H5.

Claim 4: A composition of claim 1, wherein H3 is replaced by H3A, H3B or H3C.

Many patentees have tried to use Markush claims to protect alternative compositions. Markush claims, however, are prone to the restriction requirement under 35 U.S.C. 121. For example, the original claim 6 of U.S. Pat. 7,465,466 entitled *Compositions and Methods for Prostate and Kidney Health and Disorders, an Herbal Preparation *was drafted to recite

'A Method comprising:

identifying a subject with a kidney disorder and

establishing a regimen for administering a composition comprising an aliquot of

the *Herba Epimedii *and

an aliquot of at least three supplemental herbs selected from the group of *Fructus Rosae Laerigatae, Fructus Rubi, Fructus Psoralea, Radix Morindae offcinalis, Fructus Schisandrac Chinensis, Fructus Ligustri Lucidi, Semen Cuscutae *and *Radix Astragali*' [7].

This claim was rejected and the examiner requested the applicant to select a species of the herbs. The final claim was subsequently amended to be 'A method comprising

identifying a human subject with a kidney disorder and

establishing a regimen for administering a composition to the subject, the

composition formulated to restore stasis in the kidney comprising

an aliquot of the *Herba Epimedii *and

an aliquot of each of supplemental herbs selected from the group consisting of *Fructus Rosae Laerigatae, Fructus Rubi, Fructus Psoralea, Radix Morindae offcinalis, Fructus Schisandrac Chinensis, Fructus Ligustri Lucidi, Semen Cuscutae *and *Radix Astragali*' [7].

While the patent was eventually issued, the scope of the patent was much narrower than intended.

The restriction requirement may be bypassed if a functional term is used to 'label' the interchangeable herbs in a group. The United States Patent and Trademark Office (USPTO) would not refuse to examine a claim that contains alternative language unless the subject matter of the claim lacked a 'unity of invention' [8]. The USPTO currently considers the 'unity of invention' to exist in a Markush group where the members of the group (1) share a common utility and (2) share a substantial feature essential to that utility [9]. Therefore, a functional language or generic terminology may be used to describe a group as sharing a 'common utility' to meet the 'unity of invention' requirement. For example,

Claim 1 U.S. Pat. 6,280,751 for 'essential oil' recites that 'a medicinal or cosmetic composition for oral administration comprising at least one essential oil in combination with at least one spice selected from the group consisting of *Acacia Catechu, Acanthopanax, Gracilistylus, Caesalpinia Sappan, Epimedium Spinosa, Paeonia lactiflora, Paeonia obovata, Atractylodes macrocephala, Glycyrrhiza uralexisis, Glycyrrhiza glabra, Lycium chinense, Nauclea rhyncholphylla, Cinnainomum cassia, Astragalus membranaceus, Scutellaria, baicalensis, Schizonepeta tenuifolia, Ephedra sinica, Ophiopogon japonicus, Paeonia suffruticosa, Artemisia annua, Aretemisia apiacea, Panax notoginseng, Cornus officinalis, Acorrius gramineus, Reluhania glutinosa, Gastroidia elata, Asparagus cochiichinensis, Cuscuta chinensis, Schizandra chinensis, Schizandra spenanthera, Magnolia liliflora, Epimediumbrevicomum, Epimedium grandiflorun, Epimedium brevicomum, Epimedium grandiflorun, Epimedium sagittatum, Houttuynia cordata, Polygala tenuifolia *and *Perilla frutescents *and *Aloe vera *extract.

Claim 2 recites that 'a medical or cosmetic composition according to claim 1, wherein the essential oil is selected from the group consisting of *bergamot, chamomile german, chamomile maroc, chamomile roman, cinnamon zeylanicum, clove buds, eucalyptus globules, frankincense, fennel, hyssop, juniper, lemon grass, mountain savory, niaouli, red thyme, rosemary, rose geranium, tagestes *and *ylang ylang*.'

Both claims 1 and 2 are allowable despite the long list of herbs enumerated in the Markush claim possibly because the alternative herbs belong to two functional groups, namely 'spice' and 'essential oil.'

#### Protection of multiple fufang applications

In Chinese medicine, a *fufang *formula may be used to treat a range of diseases, the philosophy of which is in stark contrast with Western medicine. Claims that a *fufang *prescription is effective to treat multiple diseases often impede a patent application. These claims are often rejected on the grounds of 35 U.S.C. 112 as 'lack of enablement.'

Under these circumstances, a patent application may be divided into various medical targets for each herbal composition. In a hypothetical example, a *fufang *formula is effective to quickly heal skin damage caused by trauma, burning or fungal infection. The herbal composition exhibits strong antibacterial and antifungal effects both *in vivo *and *in vitro*. In this case, several separate patents should be filed for each therapeutic category. One patent should emphasize the antibacterial effects. Another patent should emphasize the applications of the herbal composition for treating skin infections caused by fungi pathogens (e.g. athletes' foot).

In an actual case, an herbal medicine company discovered that a *fufang *formula reduced inflammation. In particular, the herbal formula inhibits Cyclooxygenase (COX) which is an enzyme-protein complex associated with inflammation. A number of diseases and/or symptoms are associated with the inflammation reactions controlled by COX isoenzymes, such as prostate cancer, prostatic intraepithelial neoplasia and glioblastoma (brain tumor). The inventors of the formula obtained a series of patents, of which U.S. Pat. 6,387,416 claims a method of using the composition to reduce inflammation; U.S. Pat. 7,067,159 claims a method of treating prostate cancer; U.S. Pat. 7,070,816 claims a method of treating prostatic intraepithelial neoplasia and precancerous cellular proliferations within prostatic ducts, ductiles and acini; U.S. Pat. 7,622,142 claims a method of using the same (or similar) composition to treat brain tumor.

A powerful patent protection for a *fufang *formula may be obtained as long as the chemical structures of its major active ingredients are identified, however, this is extremely difficult to achieve as *fufang *is processed from a mixture of herbs with hundreds of thousands of compounds. To this date, most successfully obtained patents for *fufang *formulae are those protecting their herbal compositions and formulations.

## Conclusion

A powerful patent protection for an herbal formula may be obtained when the chemical structures of each active ingredient are identified and patented. While this is a relatively easy task for *danfang *formulae, it remains a very daunting task for *fufang *formulae. As a result, the current patents for *fufang *formulae protect only the traditional herbal formulations. More efforts from the research community are needed to protect the various herbal combinations as well as the multiple applications of *fufang *formulae.

## Abbreviations

CM: Chinese medicine; GC: gas chromatography; HPLC: performance liquid chromatography; MS: mass specturoscopy; Pat: Patent; US: United States; USPTO: United States Patent and Trademark Office; COX: Cyclooxygenase

## Competing interests

The authors declare that they have no competing interests.

## Authors' contributions

AC supervised the study and revised the manuscript. XW conducted the research and drafted the manuscript. Both authors read and approved the final version of the manuscript.

## References

[B1] The State Council of the People's Republic of China Regulations on Protection of Traditional Chinese Medicines 1992 PRC: State Council Decree No. 106

[B2] Koopsen C Young C Integrative Health - A Holistic Approach for Health Professionals 2009 Sudbury: Jones and Bartlett Publishers, LLC

[B3] Hsiao JIH Patent protection for Chinese herbal medicine product invention in Taiwan J World Intellect Prop 2007 10 1 1 21 10.1111/j.1422-2213.2007.00311.x

[B4] Chan K Chan K, Lee H Understanding the toxicity of Chinese medicinal products The Way Forward for Chinese Medicine 2002 London: Taylor & Francis 71 91

[B5] Jiangsu College of New Medical The Encyclopedia of Traditional Chinese Medicine 1977 Zhejiang: Jiangsu College

[B6] Fu H Guo Y Li W Dou D Kang T Koike K A new angeloylated triterpenoid saponin from the husks of *Xanthoceras sorbifolia *Bunge J Nat Med 2010 64 80 84 10.1007/s11418-009-0359-z 19787422

[B7] Chou WH Compositions and methods for prostate and kidney health and disorders, an herbal preparation US Patent 7,465,466 2008

[B8] The Unites States Patent and Trademark Office Manual of Patent Examining Procedures 2010 Alexandria, USA: Patent and Trademark Office

[B9] In re Harnisch, 631 F.2d 716, 206 U.S.P.Q. 200 (C.C.P.A. 1980)

